# Algebraic Comparison of Partial Lists in Bioinformatics

**DOI:** 10.1371/journal.pone.0036540

**Published:** 2012-05-17

**Authors:** Giuseppe Jurman, Samantha Riccadonna, Roberto Visintainer, Cesare Furlanello

**Affiliations:** 1 Fondazione Bruno Kessler, Trento, Italy; 2 DISI, University of Trento, Trento, Italy; University of Minnesota, United States of America

## Abstract

The outcome of a functional genomics pipeline is usually a partial list of genomic features, ranked by their relevance in modelling biological phenotype in terms of a classification or regression model. Due to resampling protocols or to a meta-analysis comparison, it is often the case that sets of alternative feature lists (possibly of different lengths) are obtained, instead of just one list. Here we introduce a method, based on permutations, for studying the variability between lists (“list stability”) in the case of lists of unequal length. We provide algorithms evaluating stability for lists embedded in the full feature set or just limited to the features occurring in the partial lists. The method is demonstrated by finding and comparing gene profiles on a large prostate cancer dataset, consisting of two cohorts of patients from different countries, for a total of 455 samples.

## Introduction

Defining indicators for assessing ranked lists’ variability has become a key research issue in functional genomics [1]–[6], particularly when trying to warrant study reproducibility [7]. In [8], a method is introduced to detect the stability (homogeneity) of a set of ranked lists of biomarkers selected by feature selection algorithms during a molecular profiling task. This method has been used in several studies, and it is available in the Bioconductor package GeneSelector [9]. The stability indicator relies on the application of metric methods for ordered data viewed as elements of a suitable permutation group. A foundation of such theory can be found in [10], [11]. It is based on the concept of distance between two lists; in particular, the employed metric is the Canberra distance [12], [13]. The mathematical details of the stability procedure on lists of equal length are described in [14], [15]: given a set of ordered lists, the basic mechanism is to evaluate the degree of self-homogeneity of a set of ordered lists through the computation of all the mutual distances between the elements of the original set.

In practice, a reduced representation can be used by computing the Canberra distance between upper partial lists of the original complete lists, the so called top-*k* lists [16], formed by their *k* best ranked elements. However, the requirement that all lists have the same length as in [8] is a main drawback in many applications. Complete lists all share the same elements, with only their ordering being different; when considering partial top-*k* lists, the same *k* initial elements must be chosen for all sublists [17], [18].

This is usually not the case when investigating the outcomes of profiling experiments, where the employed feature ranking method often does not produce a rank for every available feature. Instead it scores only the best performing ones, thus leading to the construction of lists with different lengths. In the top-*k* list setting ranked lists are truncated in a selection procedure and their length *k* is not the same for all lists. Furthermore, rank positions are not available for all the input features. In the rank aggregation literature this phenomenon is discussed under the notion of space differences [18]–[20]. Some work towards partial lists comparison has appeared in the literature, both for general contexts [21] and more focussed on the gene ranking case [22]–[24], but they all consist of set-theoretical measures.

In the present work we propose an extension of the method introduced in [8], by computing a distance for two lists of different lengths, defined within the framework of the metric methods for permutation groups. The Canberra distance is chosen for compatibility with [8] and for further technical reasons detailed in the method description. The problem of how to select the list length is not addressed here: for a data-driven stochastic approach see [17], [25], [26] and subsequent works. The extension is again developed in the framework of permutation groups, where subsets of permutations with constraints are considered. The key formula can be split into two main components: one that addresses the elements occurring in the selected lists, and the second one considering the remaining elements of the full set of features the experiment started from. In particular, this second component is independent from the positions of the selected elements in the lists: neglecting this part, a different stability measure (called the core component of the complete formula) is obtained.

Applications and discussions of the described methods for either the complete or the partial list case can be found in [27]–[34]. Meta-analysis studies can particularly benefit from this novel tool: although it is common to have a rather small number of replicates [20], nowadays the available computing power is making studies with large numbers of replicates more and more feasible. In these settings, the quantitative assessment of list differences is crucial. Examples include MAQC-II initiative, where more than 30,000 models were built [35] for dealing with 13 tasks on 6 datasets, or the comparative study [36] where effects of 100 bootstrap replicates were assessed for 6 combinations of classifiers and feature selection algorithms on synthetic and breast cancer datasets.

After having detailed the algorithm, we discuss applications to synthetic and genomics datasets and different machine learning tasks. The described algorithm is publicly available within the Python package mlpy [37] (http://mlpy.fbk.eu) for statistical machine learning.

## Materials and Methods

### Introduction

The procedure described in [8] is composed of two separate parts, the former concerning the computation of all the mutual distances between the (complete or partial) lists, and the latter the construction of the matrix starting from those distances and the indicator coming from the defined matrix. This second phase is independent from the length of the considered lists: the extension shown hereafter only affects the previous step, *i.e.* the definition of the dissimilarity measure.

The original algorithm and its extension rely on application of metric methods for ordered data viewed as elements of a suitable permutation group: foundations of such theory can be found in [10], [11], [38], [39] and it is based on the concept of distance between two lists. In particular, the employed metric in the previous work is the Canberra distance [12], [13] and the same choice is also adopted in the present work for consistency and to ensure that the original method and the introduced novel procedure coincide on complete lists.

Full mathematical details of the original procedure are available in [14], [15].

### Notations

As in the original paper, we adopt as a working framework the formalism and notation of symmetric group theory. No theoretical result from group theory will be needed, as combinatorics will be mostly used throughout the present section.

Let 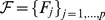 be a set of *p* features, and let *L* be a ranked list consisting of *l* elements extracted (without replacement) from 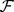. If 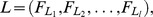 let 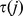 be the rank of 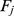 in *L* (with 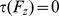 if 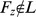) and define 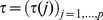 the dual list of *L*. Consider the set 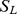 of all elements of the symmetric group 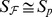 on 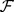 whose top-*l* sublist is 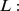 then 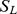 has 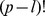 elements, corresponding to all the 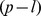 possibilities to assign the 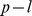 elements not in the top-*l* list to the bottom 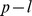 positions.

Finally, let 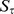 be the set of all the dual lists of the elements in 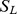: if 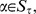 then 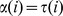 for all indexes 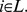 Thus 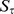 consists of the 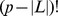 (dual) permutations of 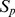 coinciding with 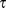 on the elements belonging to 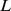. Furthermore, note that 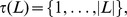 so that the relabeling 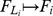 shows the isomorphism between 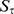 and 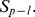


### Shorthands

If 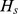 is used to denote the *s*-th harmonic number defined as 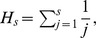 then we can define some useful shorthands:
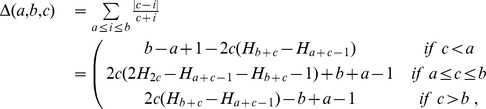
and



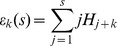


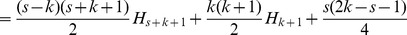


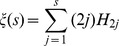


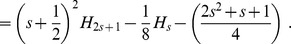



Finally,
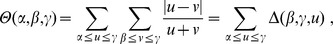
with 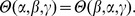 Details on harmonic numbers can be found in [40], while some new techniques for dealing with sums and products of harmonic numbers are shown in [41]–[49].

### Canberra Distance on Permutation Groups

Originally introduced in [12] and later redefined by the same authors in [13], the Canberra distance as a metric on a real line is defined as.
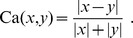



Its extension to real-valued vectors 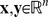 is again included in [13] and reads as follows:
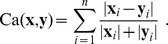



This metric can be naturally extended to a distance on permutation groups: for 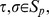 we have.
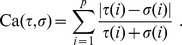



The key property for the bioinformatics applications motivating the choice of the Canberra distance is that this metric attaches more importance to changes near the beginning of a list than to later differences. Clearly, the same property belongs to other functions (*e.g.*, the difference of the logarithm of the ranks), and probably similar results as those we are discussing here can be achieved by employing different choices. We choose the Canberra distance because it has been already published in literature, it has a simple definition, satisfactory behaviour on synthetic data was shown in [8], and exact computations are available for important indicators (average variance, maximum value and argument). Finally, we chose to linearly sum terms instead of using different norms such as 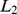 as in the original version proposed by the authors of the Canberra distance [12], [13].

The expected (average) value of the Canberra metric on the whole group 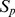 can be computed as follows, where 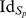 is the identity element of the permutation group 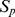 (the identical permutation):
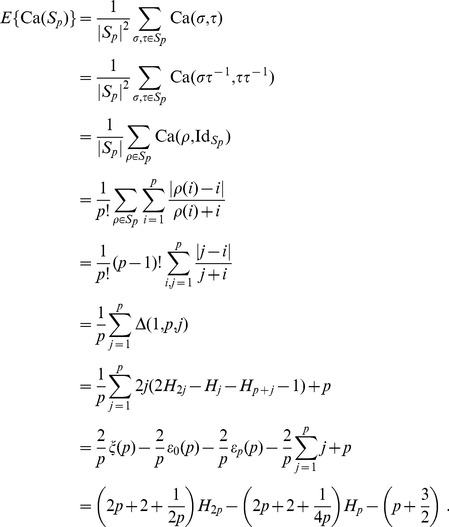
(1)


In Eq. (1), the identity.
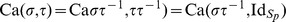
follows straightforwardly from the right-invariance of the Canberra distance as a metric on permutation groups, while the identity



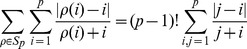
is motivated by the combinatorial observation that, for each 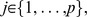 there are exactly 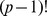 permutations 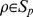 with 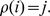


By Euler’s approximation 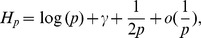 where 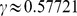 is the Euler-Mascheroni constant, the exact formula in Eq. (1) can be approximated up to terms decreasing to zero with *p* by the expression.
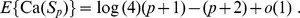



In his paper [50], Hoeffding proved four Theorems where he stated a sufficient condition for the distribution of a metric on the whole permutation group to be asymptotically normal. As shown in Result R5 of [14], this condition is satisfied by the Canberra distance, thus asymptotical normality on 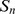 follows and therefore it is meaningful to define a stability indicator on a set of lists as the average of all mutual Canberra distances between each pair of lists in the set.

### Canberra Dissimilarity Measure on Partial Lists

As originally introduced in [8], given two complete lists 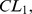 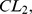 we define the Canberra distance between them as.
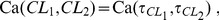
(2)where 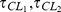 are the corresponding permutations, which are unique.

Uniqueness of matching permutations does not hold anymore when dealing with partial lists, where many permutations share the same top sublist *L*. A suitable function *f* has to be used to convey the information coming from all possible mutual distances between corresponding permutations into a single figure.

If 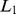 and 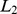 are two (partial) lists of length respectively 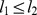 whose elements belong to 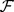, and *d* is a distance on permutation groups, we define a dissimilarity measure between 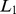 and 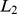 as
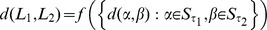


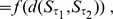
for *f* a function of the 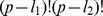 distances 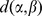 such that on a singleton *t*, 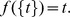 The map *d* is symmetric but, if *L* is not complete, 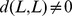 for a generic function *f*, since the contributions coming from the unselected features are taken into account, and thus *d* is not a metric. On the other hand, if 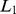 and 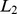 are complete lists, the above definition coincides with the usual definition of distance between complete lists given in [8]. Moreover, *d* being a dissimilarity measure, the smaller the value the more similar the compared lists.

Motivated by the fact that many distances for permutation groups are asymptotically normal [50], proven for the Canberra distance in [14], [15], a natural choice for the function *f* is the mean:
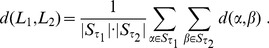
(3)


**Figure 1:**
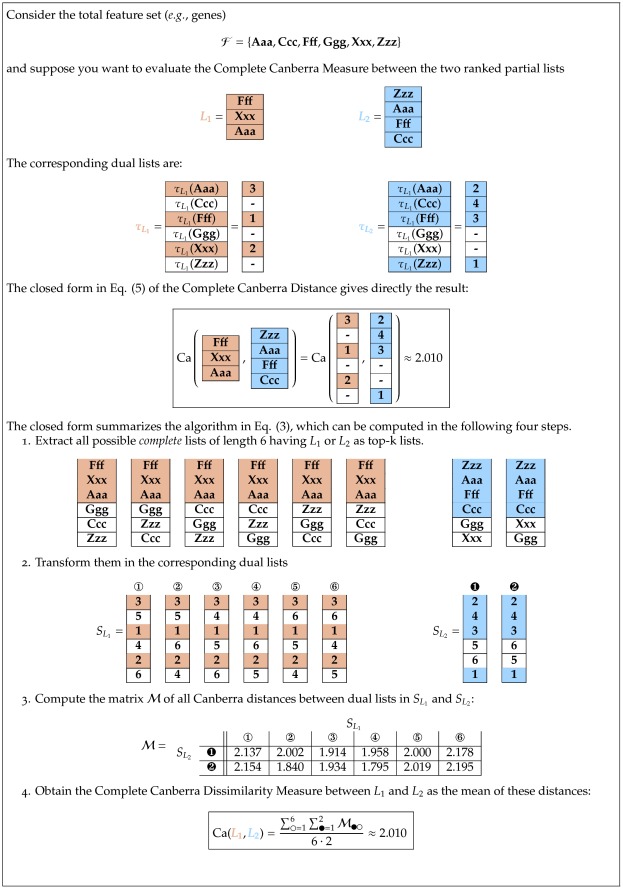
Operational steps in computing the Complete Canberra Dissimilarity Measure between two partial lists. Example on two lists of length 3 and 4 on an alphabet of 6 features, by the closed form Eq. (5) and through the open formula Eq. (3).

We point out again that this definition differs from Eq. (2), first introduced in [8], because the relation between the size of actually used features and the size of the original feature set is now taken into account here. In Fig. 1 we present a complete worked out example of the operational pipeline needed to compute 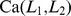 on two partial lists.

Consider the decomposition of the set 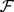 into the three disjoint sets, ignoring the rank of the features: 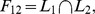 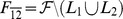 and 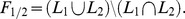 Then, if 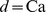 is the Canberra distance and 
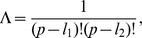
 the Eq. (3) can be split as follows into three terms:
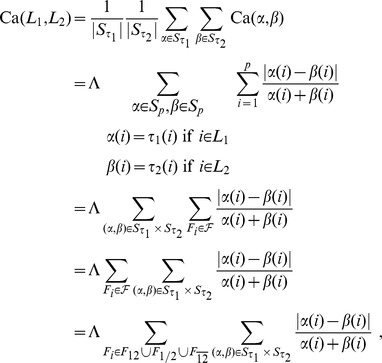
and thus



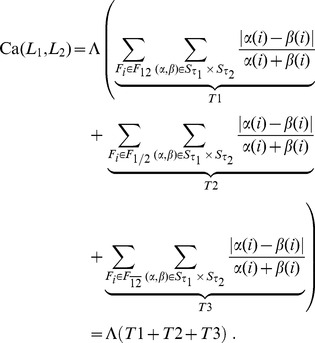
(4)We call Eq. 4 the Complete Canberra Measure between 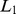 and 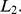 The three terms in brackets can be interpreted respectively as:

**T1** is the component computed over the features appearing in both lists 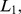 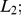


**T2** takes care of the elements occurring only in one of the two lists;

**T3** is the component concerning the elements of the original feature set appearing neither in 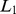 nor in 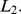


Expanding the three terms T1, T2, T3 a closed form can be obtained, so that the Complete Canberra Measure between partial lists is defined as.
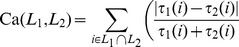


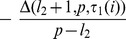
(5)

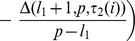


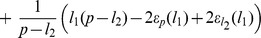


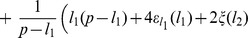


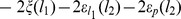


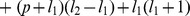


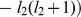


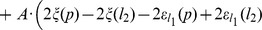


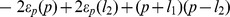


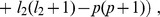
where 
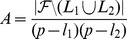
.

The availability of a closed form (5) for Eq. (4) allows calculating the dissimilarity measure between 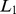 and 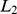 without looping through all possible pairs of complete lists with 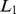 or 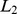 as top-*k* lists, with a consistent benefit in terms of computing time.

The sum generating the term T3 in Eq. (4) runs over the subset 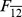 collecting all elements of the original feature set which do not occur in any of the two lists. Thus this part of the formula is independent from the positions of the elements occurring in the partial lists 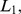 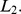 By neglecting this term, we obtain the Core Canberra Measure, defined in the above notations as.
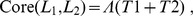
that is, the sum of the components of the Complete Canberra Measure depending on the positions of the elements in the considered partial lists. In terms of closed form, this corresponds to setting 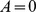 in Eq. (5) in the definition of Complete Canberra Measure.

Throughout the paper, the values of both instances of the Canberra Measure are normalized by dividing them by the expected value 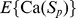 on the whole permutation group 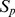 reported in Eq. (1).

A set of random (complete) lists have a Complete Canberra Measure very close to one, even for very small sets, as evidenced in Table 1 where we collect mean and variance over 10 replicated experiments of the normalized Canberra stability indicator for different sized sets of complete lists of various lengths. Note that, since the expected value is not the highest one, dissimilarity values greater than one can occur.

**Table 1:** Mean and variance of the Canberra stability indicator over 10 replicates for sets with 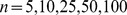 random lists with 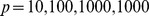 features.

n	p	Mean	Variance
5	10	0.962656	0.0047628
5	100	1.000541	0.0000557
5	1000	0.997902	0.0000141
5	10000	0.999631	0.0000031
10	10	1.012907	0.0003280
10	100	1.000535	0.0000432
10	1000	0.999643	0.0000081
10	10000	1.000165	0.0000003
25	10	0.997118	0.0003237
25	100	1.000279	0.0000108
25	1000	1.000063	0.0000020
25	10000	1.000107	0.0000001
50	10	0.998381	0.0000583
50	100	1.000145	0.0000014
50	1000	1.000122	0.0000001
50	10000	0.999999	0.0000000
100	10	0.998931	0.0000178
100	100	1.000476	0.0000004
100	1000	0.999885	0.0000002
100	10000	0.999989	0.0000000

When the number of features in 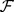 not occurring in 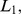 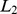 becomes larger (for instance, 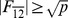), the non-core component gets numerically highly preeminent: in fact, the term T3 considers all the possible 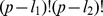 lists in 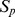 having 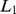 and 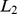 respectively as top lists; as an example, for 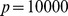 and 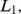 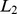 two partial lists with 100 elements, this corresponds to evaluate the distance among 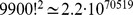 lists of elements not occurring in 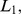 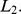 When the number of lists of unselected elements grows larger, the average distance among them would get closer to the expected value of the Canberra distance on 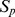 because of the Hoeffding’s Theorem.

This is quite often the case for biological ranked lists: for instance, selecting a panel of biomarkers from a set of probes usually means choosing fewer than a hundred features out of an original set of several thousands. Thus, considering the Core component instead of the Complete takes care of this dimensionality reduction of the considered problem.

**Figure 2:**
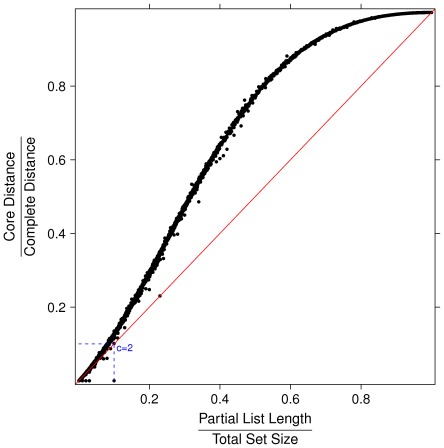
Ratio between Core and Complete measures vs. ratio between the length of partial lists and the size of the full feature set for about 7000 instances of couples of partial lists. Lists pairs have the same length and they are randomly permuted, with partial lists length ranging between 1 and 5000 and full set size ranging between 10 and 100000.

**Figure 3:**
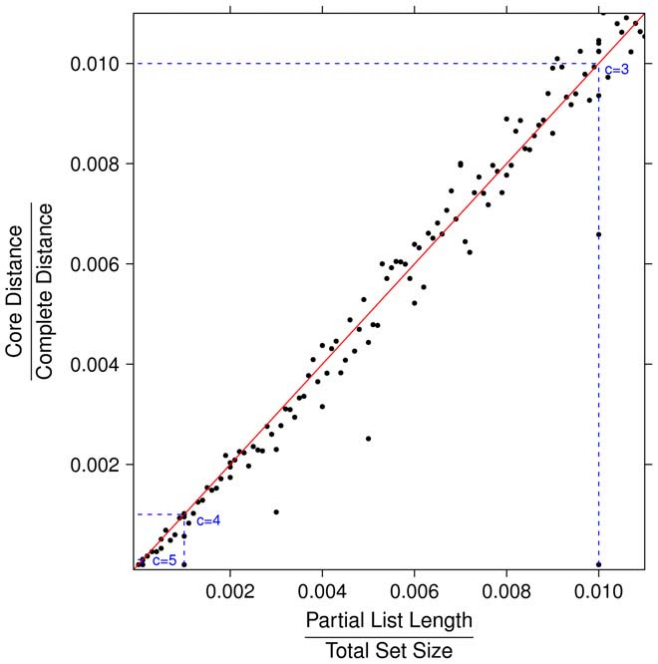
Zoom of the bottom left corner of Figure 2. Core and Complete measures are proportional when the ratio between the length of partial lists and the size of the full feature set is less than 0.15.

As an example, in Table 2 we show the values of the normalized distances of two partial lists of length 10 extracted from an original set 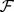 with 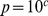 features (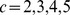), in the three cases of identical partial lists, randomly permuted partial lists (which yields average distance) and maximally distant partial lists. For the identification of the permutation maximizing the Canberra distance between lists see [14], [15]. In Fig. 2 and Fig. 3 the ratio between Core and Canberra measures are plotted versus the ratio between the length of partial lists and the size of the full feature set, for about 7000 instances of couples of randomly permuted partial lists of the same length. When the number of elements of the partial lists is a small portion of the total feature, the Complete and the Core distance are almost linearly dependent. In contrast, when such ratio approaches one the ratio between the two measures follows a different function.

**Table 2:** Core and Complete normalized Canberra dissimilarity measure for two partial lists of 10 features extracted from a set of 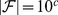 features.

Lists	Dist.	*c* = 2	*c* = 3	*c* = 4	*c* = 5
Identical	Comp.	0.692830	0.960499	0.995858	0.999583
Random	Core	0.078038	0.006368	0.000950	0.000109
Random	Comp.	0.770868	0.966867	0.996809	0.999692
Max.Dist.	Core	0.128448	0.012665	0.001265	0.000126
Max.Dist.	Comp.	0.821278	0.973164	0.997123	0.999709

The partial lists are either identical, randomly permuted (average distance) or maximally distant. The Core Measure for Identical partial lists is zero.

In summary, the Core measure is more convenient to better focus on differences occurring among lists of relatively small length. On the other hand, the Complete version is the elective choice when the original feature set is large and the partial list lengths are of comparable order of magnitude of 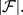


### Expansion of Eq. (4)

The three terms occurring in Eq. (4) can be expanded through a few algebraic steps in a more closed form, reducing the use of sums wherever possible.

#### T1: common features

The first term is the component of the distance computed over the features appearing in both lists 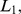 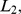 thus no complete closed form can be written. The expansion reads as follows:
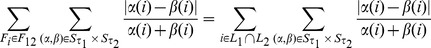


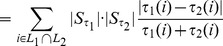


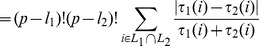


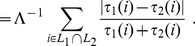



#### T2: features occurring only in one list

The second term regards the elements occurring only in one of the two lists. By defining 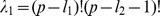 and 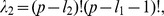 the term can be rearranged as:



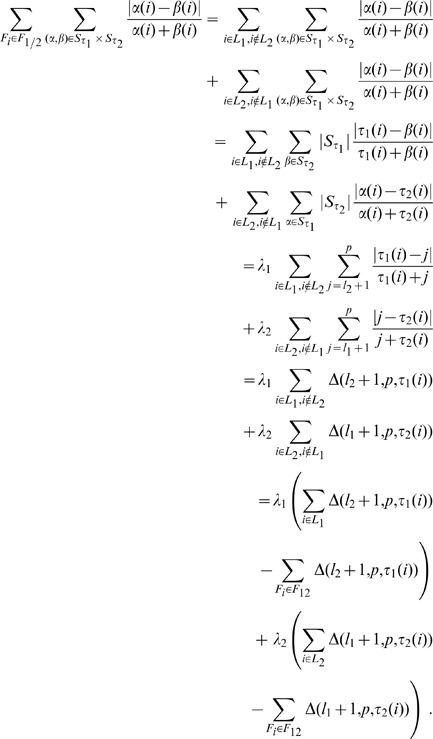



#### T3: unselected features

The last term represents the component of the distance computed on the elements of the original feature set not appearing in 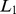 or 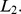 Here a complete closed form can be reached:



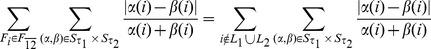


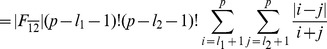


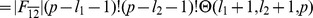


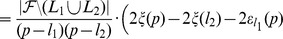


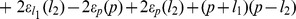


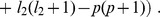



### The Borda List

To summarize the information coming from a set of lists 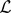 into a single optimal list, we adopt the same strategy of [8], *i.e.* an extension of the classical voting theory methods known as the Borda count [51], [52]. This method derives a single list from a set of *B* lists on *p* candidates 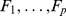 by ranking them according to a score 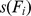 defined by the total number of candidates ranked higher than 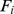 over all lists. Our extension consists in first computing, for each feature 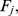 its number of extractions (the number of lists where 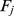 appears) 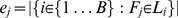 and its average position number 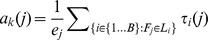 and then ranking the features by decreasing extraction number and by increasing average position number when ties occur. The resulting list will be called optimal list or Borda list. The equivalence of this ranking with the original Borda count is proved in [8].

### Implementation

Computing stability indicator for a set of *B* partial lists in a original set of *p* features has a computational cost of 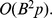 The computation of the stability indicator for partial lists is publicly available in the Open Source Python package for statistical machine learning mlpy (http://mlpy.fbk.eu) [37], since version 1.1.2. Formula (5) is used for computing both the Complete and the Core Canberra Measures. The algorithm implementation is in ANSI C to enhance efficiency, and linking to the Python framework is realized by means of the Cython interface.

## Results

We demonstrate an application of the partial list approach in functional genomics. We consider a profiling experiment on a publicly available prostate cancer dataset: the task is to select a list of predictive biomarkers and a classifier to predictively discriminate prostate cancer patients carrying the TMPRSS2-ER gene fusion. We apply the approach to compare different configurations of the learning scheme (*e.g.*, the classifier, or the ranking algorithm). In particular, the quantitative analysis of the stability of the ranked partial lists produced by replicated cross-validations is used to select the desired panel and to detect differences between the two cohorts in the dataset.

### Data Description

The Setlur Prostate Cancer Dataset was described in [53] and it is publicly available from GEO (website http://www.ncbi.nlm.nih.gov/geo, accession number GSE8402); gene expression is measured by a custom Illumina DASL Assay of 6144 probes known from literature to be prostate cancer related. Setlur and colleagues identified a subtype of prostate cancer characterized by the fusion of the 5′-untranslated region of the androgen-regulated transmembrane protease serine 2 (TMPRSS2) promoter with erythroblast transformation-specific transcription factor family members (TMPRSS2-ER). A major result of the original paper is that this common fusion is associated with a more aggressive clinical phenotype, and thus a distinct subclass of prostate cancer exists, defined by this fusion. The profiling task consists in separating positive TMPRSS2-ERG gene fusion cases from negative ones from transcriptomics signals, thus identifying a subset of probes associated to the fusion. The database includes two different cohorts of patients: the US Physician Health Study Prostatectomy Confirmation Cohort, with 41 positive and 60 negative samples, and the Swedish Watchful Waiting Cohort, consisting of 62 positive and 292 negative samples. In what follows, we will indicate the whole dataset as Setlur, and its two cohorts by the shorthands US and Sweden. The investigated problem is a relatively hard task, as confirmed also by the similar study conducted on a recently updated cohort [54].

### Predictive Biomarker Profiling Setup

**Figure 4:**
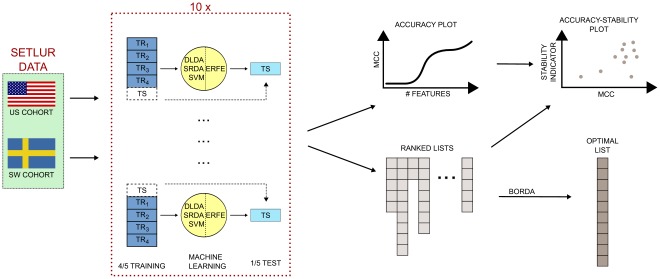
Analysis pipeline for the classifier/feature ranking methods: a 100×5-fold CV is applied separately on the two cohorts, and a set of models is build on increasing number of important features, ranked by discriminant power for the employed classifier. At the same time, the stability level of the set if derived lists is computed, and all models are evaluated on a accuracy-stability plot.

Following the guidelines of the MAQC-II study [35], a basic Data Analysis Protocol (DAP for short) is applied to both cohorts of the Setlur dataset, namely a stratified 10×5-CV, using three different classifiers: Diagonal Linear Discriminant Analysis (DLDA), linear Support Vector Machines (lSVM), and Spectral Regression Discriminant Analysis (SRDA). A workflow representation of this pipeline is shown in Fig. 4. We describe here the main characteristics of the cited algorithms.

DLDA [55] implements the maximum likelihood discriminant rule, for multivariate normal class densities, when the class densities have the same diagonal variance-covariance matrix; in this model variables are uncorrelated, and for each variable, the variance is the same for all classes. The algorithm employs a simple linear rule, where a sample is assigned to the class *k* minimizing the function 
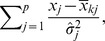
 for *p* the number of variables, 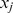 the value of the test sample *x* on gene *j*, 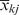 the sample mean of class *k* and gene *j*, and 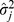 the pooled estimate of the variance of gene *j*. Although concise and based on strong assumptions (independent multivariate normal class densities), DLDA is known to perform quite well, even when the number of cases is smaller than the number of variables, and it has been successfully employed for microarray analysis in extensive studies [35]. Furthermore, a score is assigned to each feature which can be interpreted as a feature weight, allowing direct feature ranking and selection. Details can be found in [56]–[58].

lSVM [59] is an algorithm aimed at finding an optimal separating hyperplane between the classes. When the classes are linearly separable, the hyperplane is located so that it has maximal margin (*i.e.*, so that there is maximal distance between the hyperplane and the nearest point of any of the classes). When the data are not separable and thus no separating hyperplane exists, the algorithm tries to maximize the margin allowing some classification errors subject to the constraint that the total error is bounded. The coefficients of the detected hyperplane are then used as weights for feature ranking.

SRDA [60] is a member of the Discriminant Analysis algorithms family, that exploits the regression framework to improve computational efficiency. Spectral graph analysis is used for solving a set of regularized least squares problems thus avoiding the eigenvector computation. A regularization value α is the only parameter needed to be tuned. For SRDA, too, a score is assigned to each feature from which a feature weight is derived for feature ranking purposes. Details on both classification and weighting are discussed in [60], [61].

A tuning phase through landscaping (*i.e.*, testing a set of parameter values on a grid) identified 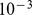 as the optimal value for the lSVM regularizer *C* on both dataset, and 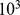 and 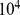 as the two values for the SRDA parameter α respectively on the US and the Sweden cohort (no tuning is needed for the DLDA classifier). Furthermore, in the lSVM case the dataset is standardized to mean zero and variance one.

As the generic feature ranking algorithm we adopt a variant of the basic RFE algorithm, described in [62]: the classifier is run on the training set and the features ranked according to their contribution to the classification. At each step, the less contributing feature is discarded and the classifier retrained, until only the top feature remains. Since RFE is computationally very costly, many alternative lighter versions appeared in literature: most of them consisting in discarding more than one feature at each step. The number of features to be discarded at each step being discarded is either fixed of determined by a function of the *n* remaining features. These alternative methods have a major drawback in the fact that they are parametric, so they ignore the structure of the resulting feature weights. The Entropy based variant E–RFE instead takes into account such weight distribution, and adaptively discards a suitable number of features after the evaluation of a entropy function: in [63] the authors show that, with respect to the original algorithm, the computational cost is considerably lower, but the resulting accuracy is comparable. Moreover, when the number of features is reduced to less than a shortlist length *z*, E-RFE reverts back to RFE: in this case, 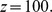 Here the E–RFE ranking algorithm is run on the training portion of the cross-validation split and classification models with increasing number of best ranked features are computed on the test part.

### Measuring Classifier Performance

Classifier performance evaluation is assessed by the Matthew Correlation Coefficient (MCC) [64] defined in Eq. (6)) and the Area Under the ROC Curve (AUC), as in Eq. (7). Measures are averaged over the cross-validation replicates, and reported for different feature set sizes. AUC is computed by Wilcoxon-Mann-Whitney formula Eq. (7) to extend the measure to binary classifiers. In [65]–[67] the equivalence with other formulations is shown: in particular, it is proved that the Wilcoxon-Mann-Whitney formula is an unbiased estimator of the classical AUC. The two performance metrics adopted have been chosen because they are generally regarded as being two of the best measures in describing the confusion matrix (see Table 3) of true and false positives and negatives by a single number. MCC’s range is 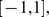 where MCC = 0 corresponds to the no-information error rate, which is, for a dataset with *P* positive samples and *N* negative samples, equivalent to 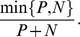 MCC = 1 is the perfect classification (FP = FN = 0), while MCC = −1 denotes the worst possible performance TN = TP = 0.

**Table 3:** Confusion matrix for a binary problem T/F: true/false; TP+FN: all positive samples, TN+FP: all negative samples.

		Actual value	
		Positive	Negative
**Predicted**	**Positive**	TP	FP
**value**	**Negative**	FN	TN



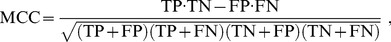
(6).
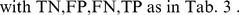





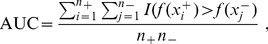
(7).
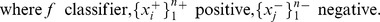



### Profiling Accuracy and Stability

In Tabless 4 and 5 we report the performances on lSVM and SRDA on discrete steps of top ranked features ranging from 5 to 6144, with 95% bootstrap confidence intervals; for comparison purposes we also report AUC values in Table 6. For the same values *k* of the feature set sizes, the Canberra Core Measure is also computed on the top-*k* ranked lists as produced by the E–RFE algorithm: the stability is also shown in the same tables. DLDA automatically chooses the optimal number of features to use in order to maximize MCC by tuning the internal parameter 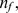 starting from the default value 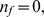 thus it is meaningless to evaluate this classifier on a different feature set size. In particular, DLDA reaches maximal performances with one feature. This is the same for all replicates, DAP2_5229, leading to a zero stability value: the resulting MCC is 0.26 (CI: (0.18, 0.34)) and 0.16 (CI: 0.12, 0.19) respectively for the US and the Sweden cohort. As a reference, 5-CV with 9-NN, which has higher performance than 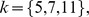 has MCC 0.36 on both cohorts with all features.

**Table 4:** MCC and Core Canberra values for the two Setlur datasets for lSVM classifiers.

step	US			Sweden		
	MCC	CI 95%	Core	MCC	CI 95%	Core
1	0.00	(0.00;0.00)	0.00	0.00	(0.00;0.00)	0.00
5	0.00	(0.00;0.00)	0.00	0.00	(0.00;0.00)	0.00
10	0.00	(0.00;0.00)	0.01	0.00	(0.00;0.00)	0.01
15	0.00	(0.00;0.00)	0.01	0.00	(0.00;0.00)	0.01
20	0.00	(0.00;0.00)	0.02	0.00	(0.00;0.00)	0.02
25	0.00	(0.00;0.00)	0.02	0.00	(0.00;0.00)	0.02
50	0.00	(0.00;0.00)	0.04	0.00	(0.00;0.00)	0.04
100	0.00	(0.00;0.00)	0.08	0.00	(0.00;0.00)	0.08
1000	0.51	(0.47;0.56)	0.52	0.08	(0.05;0.12)	0.52
5000	0.53	(0.49;0.58)	0.88	0.23	(0.20;0.27)	0.91
6144	0.53	(0.49;0.58)	0.59	0.24	(0.20;0.27)	0.62

**Table 5:** MCC and Core Canberra values for the two Setlur datasets for SRDA classifiers.

step	US			Sweden		
	MCC	CI 95%	Core	MCC	CI 95%	Core
1	0.67	(0.61;0.72)	0.00	0.40	(0.36;0.43)	0.00
5	0.55	(0.51;0.60)	0.00	0.30	(0.26;0.34)	0.00
10	0.57	(0.53;0.62)	0.01	0.33	(0.29;0.36)	0.01
15	0.57	(0.53;0.62)	0.01	0.36	(0.32;0.39)	0.01
20	0.57	(0.53;0.62)	0.02	0.39	(0.34;0.43)	0.02
25	0.57	(0.52;0.61)	0.02	0.43	(0.39;0.47)	0.02
50	0.61	(0.57;0.65)	0.04	0.44	(0.41;0.47)	0.04
100	0.59	(0.54;0.64)	0.08	0.44	(0.40;0.48)	0.08
1000	0.50	(0.9;0.55)	0.52	0.47	(0.43;0.50)	0.51
5000	0.51	(0.46;0.56)	0.89	0.46	(0.43;0.50)	0.84
6144	0.51	(0.46;0.56)	0.60	0.46	(0.42;0.49)	0.52

**Table 6:** AUC values for the two Setlur datasets for SRDA and lSVM classifiers.

step	SRDA				lSVM			
	US		Sweden		US		Sweden	
	AUC	CI 95%	AUC	CI 95%	AUC	CI 95%	AUC	CI 95%
1	0.87	(0.84;0.89)	0.79	(0.77;0.80)	0.87	(0.84;0.89)	0.51	(0.44;0.58)
5	0.83	(0.81;0.85)	0.79	(0.77;0.80)	0.84	(0.82;0.86)	0.78	(0.76;0.81)
10	0.86	(0.84;0.88)	0.80	(0.79;0.82)	0.86	(0.84;0.88)	0.79	(0.78;0.81)
15	0.88	(0.86;0.89)	0.82	(0.81;0.83)	0.87	(0.85;0.89)	0.80	(0.79;0.82)
20	0.88	(0.86;0.90)	0.83	(0.81;0.84)	0.88	(0.86;0.90)	0.81	(0.80;0.82)
25	0.89	(0.87;0.91)	0.84	(0.82;0.85)	0.89	(0.87;0.91)	0.81	(0.79;0.82)
50	0.90	(0.89;0.92)	0.84	(0.83;0.86)	0.90	(0.88;0.92)	0.82	(0.80;0.83)
100	0.90	(0.88;0.92)	0.85	(0.84;0.86)	0.90	(0.88;0.91)	0.82	(0.81;0.84)
1000	0.86	(0.85;0.88)	0.83	(0.81;0.84)	0.86	(0.84;0.88)	0.83	(0.82;0.85))
5000	0.86	(0.84;0.88)	0.82	(0.81;0.84)	0.85	(0.83;0.87)	0.83	(0.81;0.84))
6144	0.86	(0.84;0.88)	0.82	(0.81;0.84)	0.85	(0.83;0.87)	0.83	(0.81;0.84))

**Figure 5:**
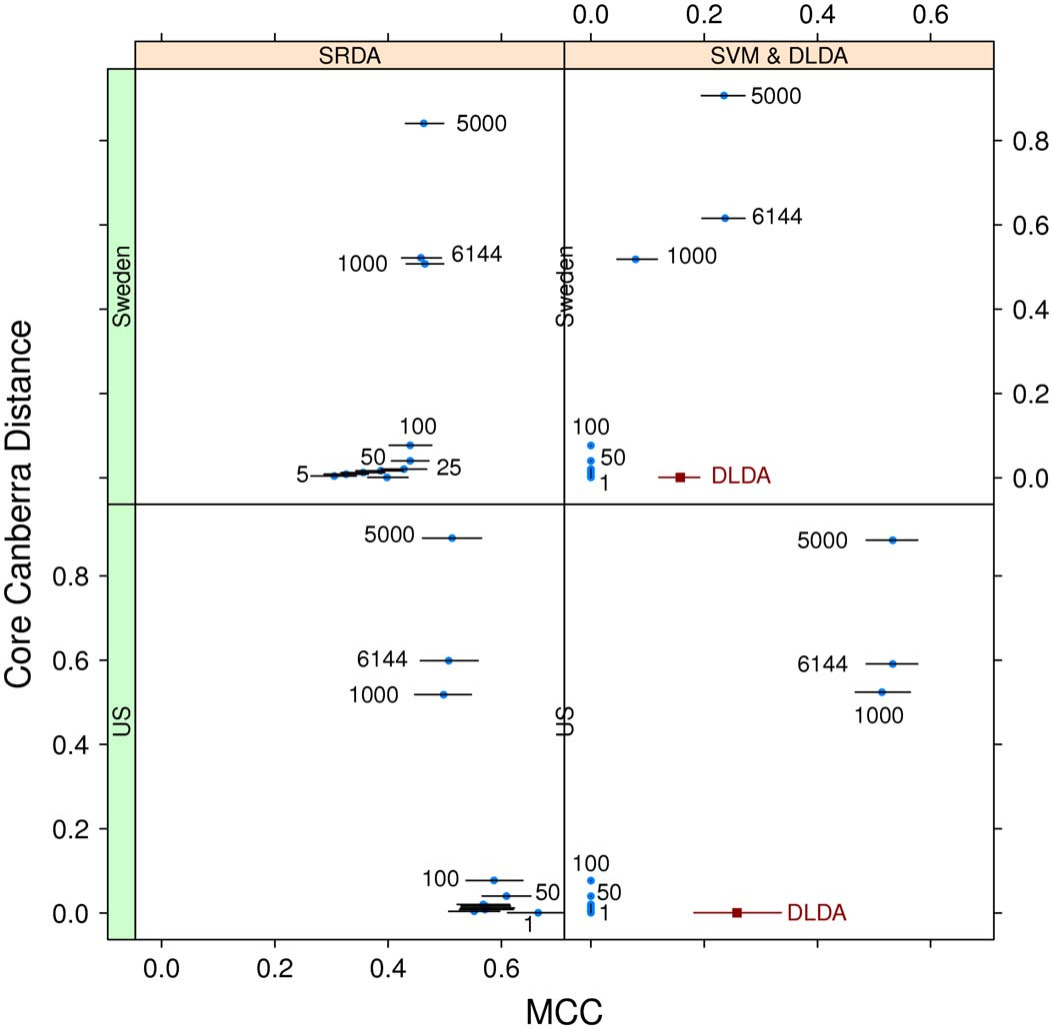
MCC and Canberra Core values on the two Setlur datasets computed by using the SRDA, lSVM, and DLDA models. Each point indicates a model with a fixed number of features, marked above the corresponding 95% Student bootstrap CI line.

**Figure 6:**
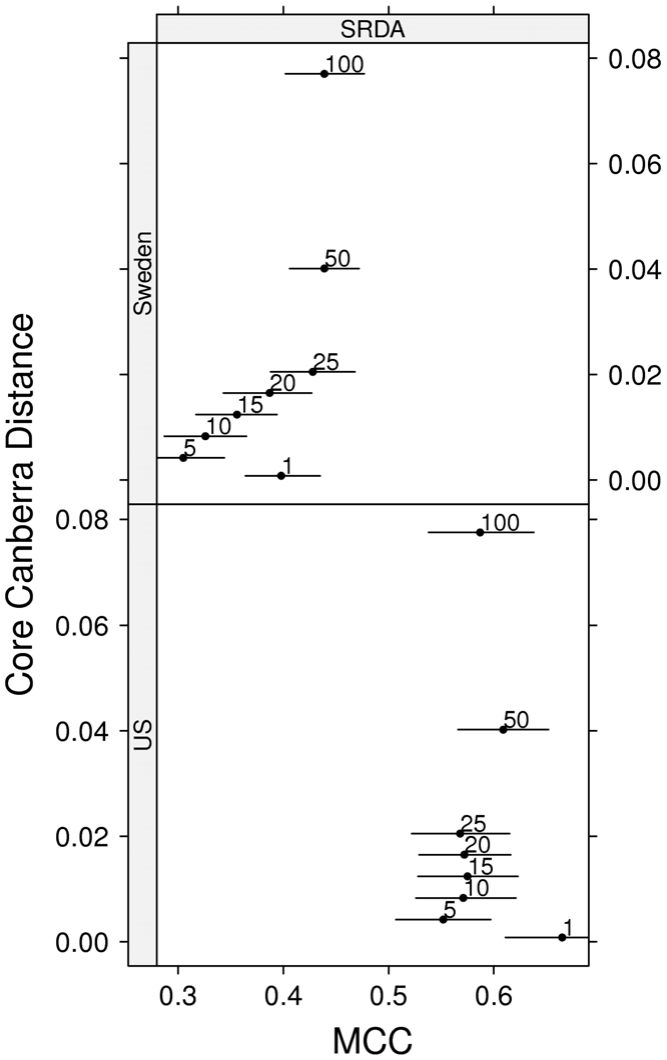
Zoom of MCC and Canberra Core values computed by using the SRDA, lSVM, and DLDA models on the two Setlur datasets. Each point indicates a model with a fixed number of features, marked above the corresponding 95% Student bootstrap CI line.

All results are displayed in the performance/stability plots of Fig. 5 and 6. These plots can be used as a diagnostic for model selection to detect a possible choice for the optimal model as a reasonable compromise between good performances (towards the rightmost part of the graph) and good stability (towards the bottom of the graph). For instance, in the case shown we decide to use SRDA as the better classifier, using 25 features on the Sweden cohort and 10 on the US cohort: looking at the zoomed graph in Fig. 6, if we suppose that the points are describing an ideal Pareto front, the two chosen models are the closest to the bottom right corner of the plots. The corresponding Borda optimal lists for SRDA models on the two Setlur datasets are detailed in Table 7∶5 probes are common to the two lists, and, in particular, the top ranked probe is the same. In Table 8 we list the MCC obtained by applying the SRDA and DLDA models on the two Setlur cohorts (exchanging their role as training and test set) by using the two optimal Borda lists.

**Table 7:** Borda optimal lists for SRDA models on the two Setlur datasets.

Sweden	Ranking in US	US	Ranking in Sweden
***DAP2_5229***	*1*	***DAP2_5229***	***1***
***DAP1_2857***	*5*	**DAP1_5091**	**18**
***DAP4_2051***	*3*	***DAP4_2051***	*3*
*DAP1_1759*	*13*	DAP2_1680	51
DAP1_2222	19	***DAP1_2857***	***2***
*DAP4_0822*	*44*	**DAP3_0905**	**8**
*DAP2_0361*	*403*	DAP2_5769	*77*
**DAP3_0905**	**6**	DAP4_2271	36
*DAP2_5076*	*24*	*DAP4_3958*	*44*
*DAP3_2016*	*16*	DAP4_2442	2734
DAP4_4217	497		
*DAP2_0721*	*421*		
*DAP4_1360*	*18*		
*DAP3_1617*	*15*		
DAP1_5829	529		
*DAP3_6085*	*12*		
DAP4_2180	26		
**DAP1_5091**	**2**		
DAP1_2043	1989		
*DAP4_2027*	*2227*		
*DAP4_1375*	*145*		
DAP4_5930	3455		
*DAP4_4205*	*25*		
DAP1_4950	166		
*DAP4_1577*	*283*		

In boldface, probes common to the two optimal lists. In italics, probes included in the 87-gene signature of the original paper [53]. 17 probes out of 30 are common to the 87-gene signature in [53].

**Table 8:** MCC values for SRDA and DLDA optimal models on the Setlur dataset.

Borda	Training	Test	SRDA	DLDA
US	US	Sweden	0.39	0.44
Sweden	Sweden	US	0.42	0.48
US	Sweden	US	0.48	0.63
Sweden	US	Sweden	0.51	0.9
US	Sweden	Sweden	0.39	0.9
Sweden	US	US	0.69	0.71
US	US	US	0.71	0.78
Sweden	Sweden	Sweden	0.55	0.52

**Figure 7:**
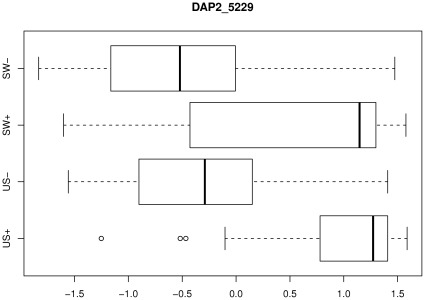
Boxplot of the DAP2_5229 expression value separately for the two Setlur datasets and the two class labels.

The probe DAP2_5229 is confirmed to have a relevant discriminative and predictive importance, by the classwise boxplots on the two cohorts of Fig. 7. As detailed in GEO and in NCBI Nucleotide DB (http://www.ncbi.nlm.nih.gov/nuccore/), its RefSeq ID is NM_004449, whose functional description is reported as “v-ets erythroblastosis virus E26 oncogene homolog (avian) (ERG), transcript variant 2, mRNA” (information updated on 28 June 2009). In Table 9 we analyse the performances obtained by a SRDA and a DLDA model with the sole feature DAP2_5229 on all combinations of US and Sweden cohort as training and test set. The high performance reached by these single feature models are supporting the claim in [68] of the global effectiveness of single-gene models in microarray studies. Finally, if we consider as the global optimal list *O* the list of all 30 distinct features given as the union of the Borda list in Table 7, obtaining for SRDA and DLDA models the performances listed in Table 9.

To check the consistency of the global list *O*, we run a permutation test: we randomly extract 30 features out of the original 6144 features, and we use as the p-value the number of times the obtained performances (DLDA models) are better than those obtained with *O*, divided by the total number 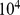 of experiments. The resulting p-values are less than 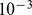 for all four combinations of using the two cohorts as training and test set, thus obtaining a reasonable significance of the global optimal list *O*. Nevertheless, if the same permutation test is run with the feature DAP2_5229 always occurring in the chosen random feature sets, the results are very different: namely, the p-value results about 0.1, thus indicating a small statistical significance of the obtained global list. These tests seem to indicate that the occurrence of DAP2_5229 plays a key role in finding a correct predictive signature.

**Table 9:** MCC values for SRDA and DLDA models with the only feature DAP2_5229 and with the global optimal list.

		SRDA		DLDA	
Training	Test	DAP2 5229	global optimal	DAP2 5229	global optimal
US	Sweden	0.47	0.47	0.49	0.48
Sweden	US	0.56	0.39	0.52	0.66
Sweden	Sweden	0.50	0.55	0.39	0.56
US	US	0.68	0.73	0.68	0.76

**Figure 8:**
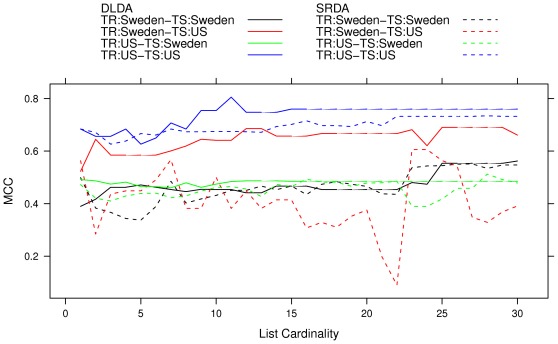
MCC for SRDA and DLDA models on increasing number of features extracted from the global list from 1 to 30 on the Setlur data.

We then performed a further experiment to detect the predictive power of *O* as a function of its length. We order the global list keeping DAP2_5229, DAP4_2051, DAP1_2857, DAP3_0905, and DAP1_5091 as the first five probes and compute the performances of a DLDA model by increasing the number of features extracted from the global list from 1 to 30. The result is shown in Fig. 8: for many of the displayed models a reduced optimal list of about 10–12 features is sufficient to get almost optimal predictive performances. A permutation test on 12 features (with DAP2_5229 kept as the top probe) gives a p-value of 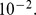


A final note: our results show a slightly better (not statistically significant) AUC in training than the one found by the authors of the original paper [53], both in the Sweden and in the US cohort. Moreover, as many as 17 out of 30 genes included in the global optimal list are member of the 87-gene signature shown in the original paper.

### Comparison with Filter Methods

The multivariate machine learning methods are usually seen as alternatives to the families of statistical univariate algorithms aimed at identifying the genes which are differentially expressed between two groups of samples. When the sample size is small univariate methods may be quite tricky, since the chances of selecting false positives are higher. Many algorithms have been devised to deal with the detection of differentially expressed genes: an important family is represented by the filter methods, which essentially consist in applying a suitable statistic to the dataset to rank the genes in term of a degree of differential expression, and then deciding a threshold (cutoff) on such degree to discriminate the differentially expressed genes.

The seven statistics considered in this experiment are Fold Change (FC) [69], Significance Analysis of Microarray (SAM) [69], *B* statistics [70], *F* statistics [71], *t* statistics [72], and mod-*F* and mod-*t* statistics [73], which are the moderated version of *F* and *t* statistics. The FC of a given gene is defined here as the ratio of the average expression value computed over the two groups of samples. All filtering statistics are computed by using the package DEDS [74] in the BioConductor extension [75] of the statistical environment R [76].

Reliability of a method over another is a debated issue in literature: while some authors believe that the lists coming from using FC ratio are more reproducible than those emerging by ranking genes according to the *P*-value of *t*-test [77], [78], others [79] point out that *t*-test and *F*-test better address some FC deficiencies (*e.g.*, ignoring variation within the same class) and they are recommended for small sample size datasets. Most researchers also agree on the fact that SAM [69], [80]–[83] outperforms all other three methods because of its ability to control the false discovery rate. Moreover, in [84] the authors show that motivation for the use of either FC or mod-*t* is essentially biological while ordinary *t* statistic is shown to be inferior to the mod-*t* statistic and therefore should be avoided for microarray analysis. In the extensive study [72], alternative methods such as Empirical Bayes Statistics, Between Group Analysis and Rank Product have been taken into account, applying them to 9 publicly available microarray datasets. The resulting gene lists are compared only in terms of number of overlapping genes and predictive performance when used as features to train four different classifiers.

**Figure 9:**
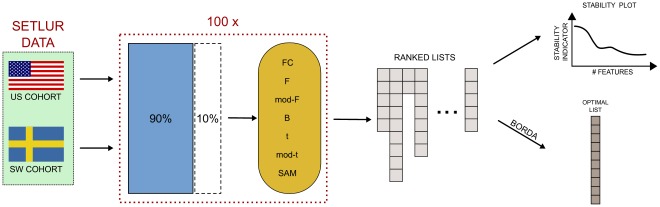
Analysis pipeline for the filtering methods: a 90%/10% split is repeated 100 times, and the selected filter method applied on the training portion. The stability indicator is then computed for the corresponding set of lists.

**Figure 10:**
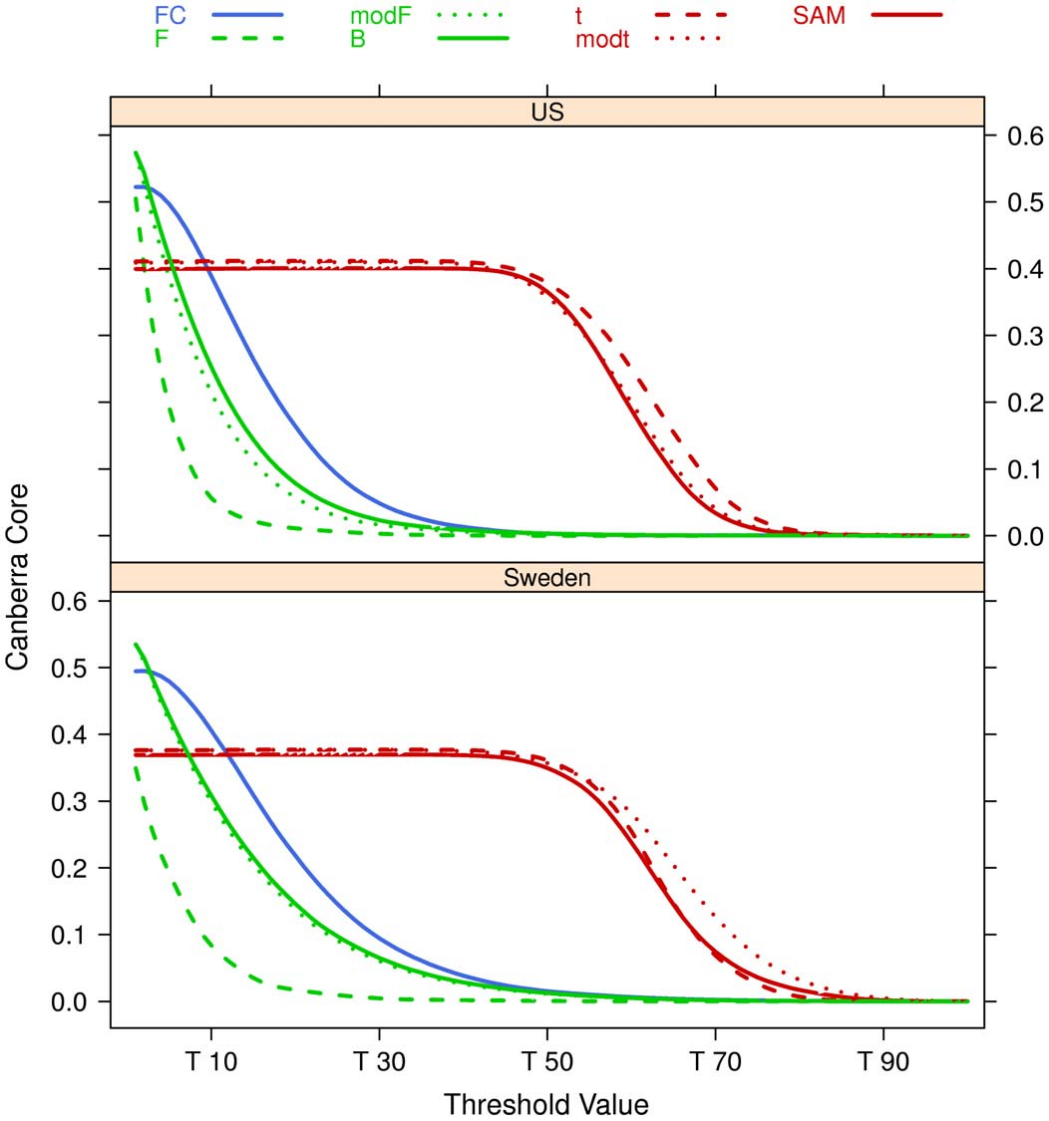
Canberra core evaluated on the Setlur dataset on B = 100 repeated filtering experiments on 90% of the data.

**Figure 11:**
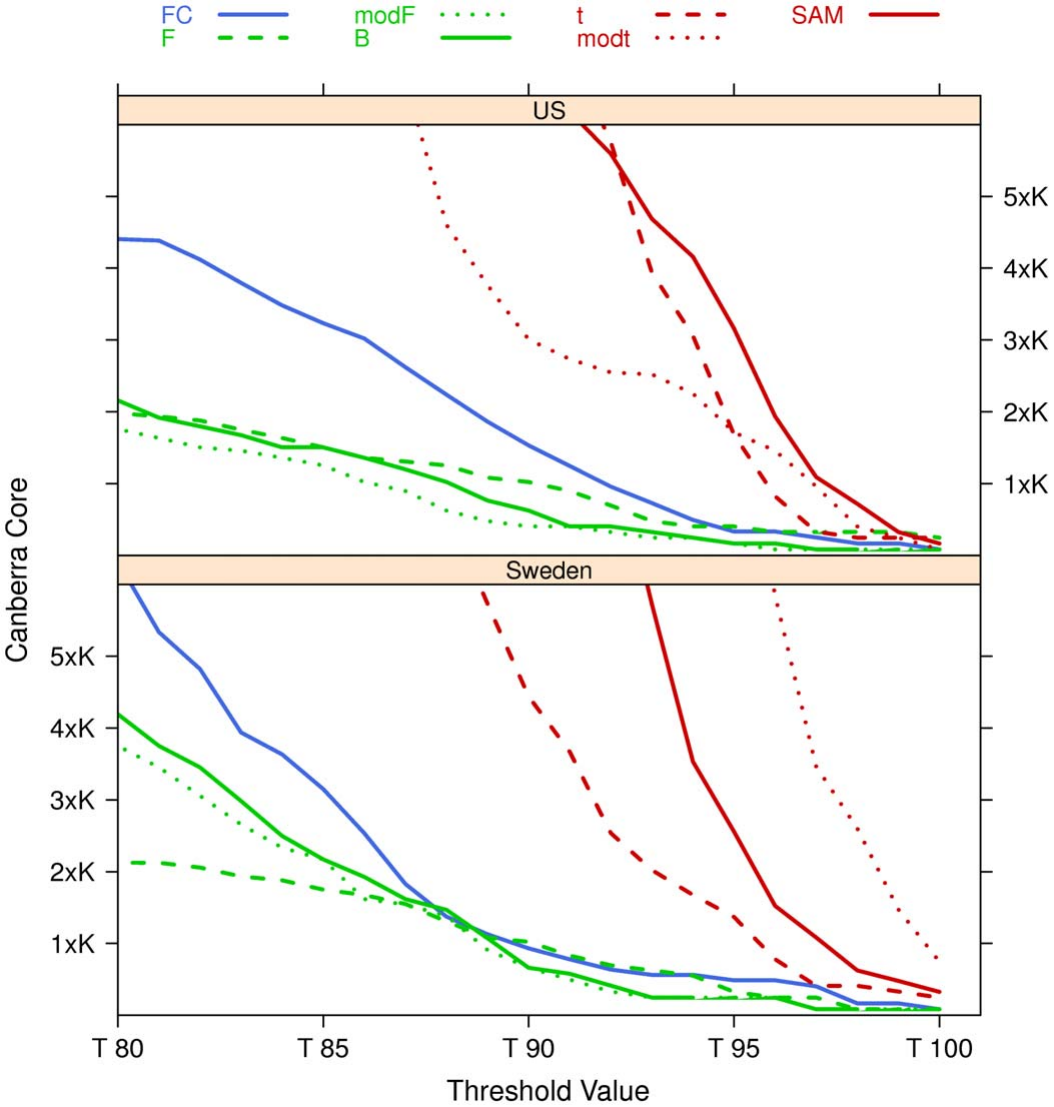
Zoom of Fig. 10 on the 80%–100% threshold zone. 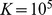
.

The seven filtering algorithms of the previous subsection are applied to the Setlur dataset by using 100 resamples on 90% of the data on both the US and Sweden cohorts separately, as shown in Fig. 9. The Canberra Core values of the lists at different values of the filtering thresholds are shown in Fig. 10, together with a zoom (Fig. 11) on the stricter constraints area: the plots highlight the different behaviours of the groups 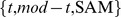 and 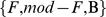 and of the singleton *FC* in both cases.

By considering a cutoff threshold of the 75% of the maximal value, we retrieve 14 sets of ranked partial lists, from which 14 Borda optimal lists are computed. In Table 10 we list the lengths of the Borda lists for each filtering method and cohort. As a rough set-theoretical comparison, we list in Table 11 the probes common to more than three filtering methods. We note that only three probes also appear in the corresponding SRDA Borda list.

**Table 10:** Length of the Borda lists for different filtering methods at 75% threshold on the Setlur dataset.

	F	FC	mod-F	mod-t	t	B	SAM
Sweden	1	17	25	759	326	28	366
US	1	3	6	208	367	7	149

**Table 11:** List of probes common to more than three filtering methods.

Sweden		US	
gene	extractions	gene	extractions
DAP2_1768	6	DAP2_4092	5
DAP1_1949	5	DAP2_5047	5
DAP1_4198	5	**DAP2_5229**	**5**
DAP1_5095	5	**DAP4_2442**	**5**
DAP2_1037	5	**DAP4_2051**	**4**
DAP2_1151	5		
DAP2_3790	5		
DAP2_3896	5		
DAP2_5650	5		
DAP3_2164	5		
DAP3_4283	5		
DAP3_5834	5		
DAP4_1974	5		
DAP4_2316	5		
DAP4_4178	5		
*(13 genes)*	4		

In boldface, the three probes appearing in the corresponding SRDA Borda list. For the Swedish cohort, 13 genes are extracted four times.

**Figure 12:**
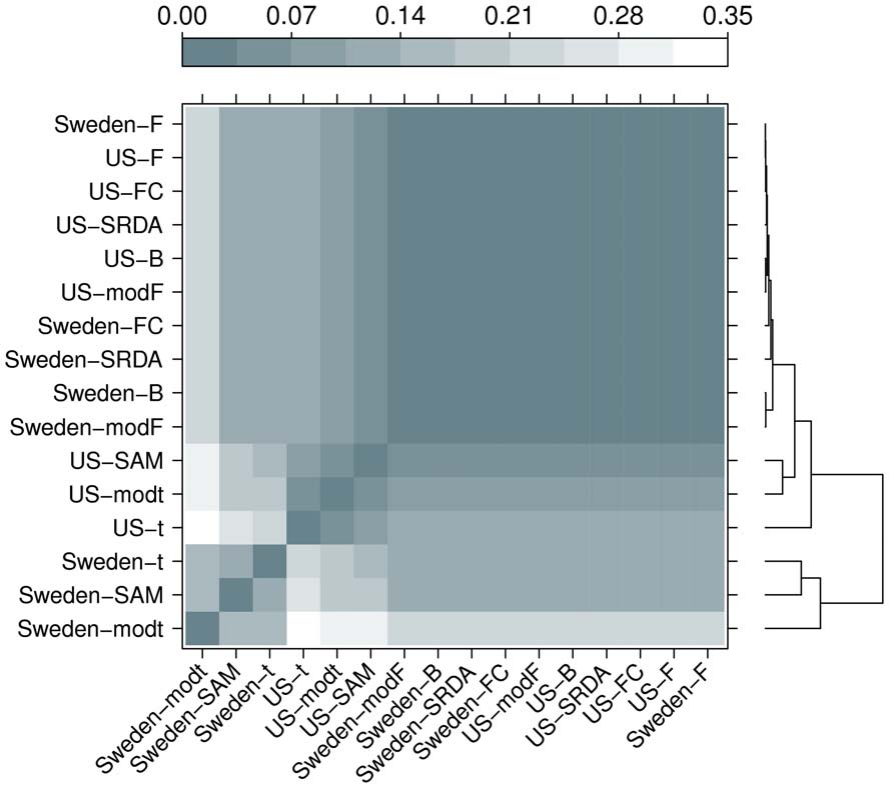
Levelplot of the values computed on the lists produced by filtering methods (75% threshold) and SRDA models with Complete Canberra Measure computed on their Borda lists.

**Figure 13:**
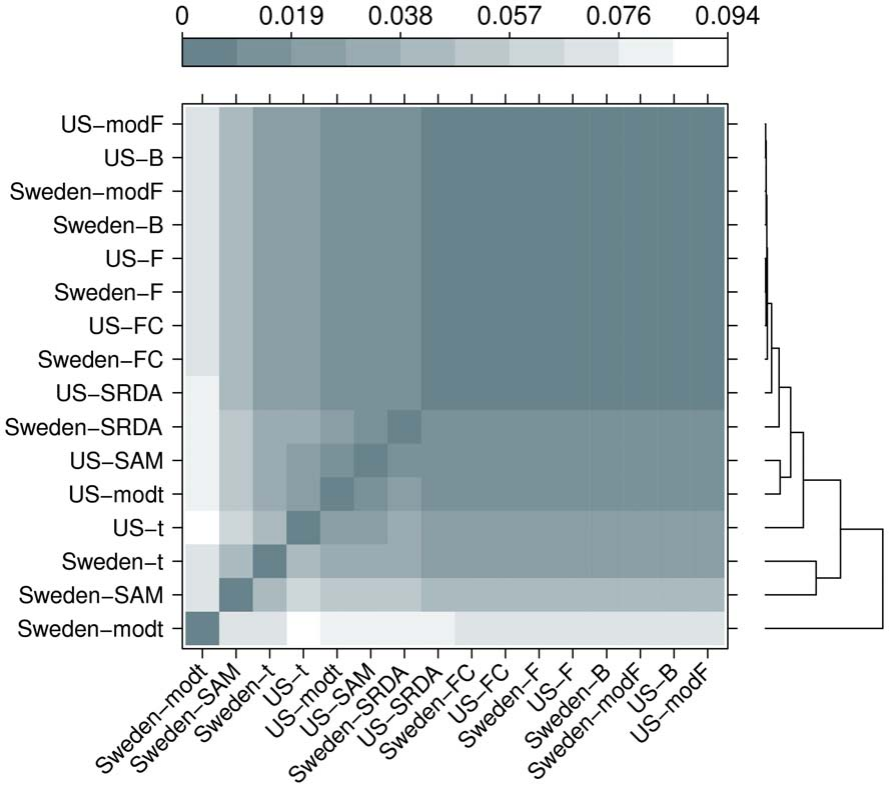
Levelplot of the values computed on the lists produced by filtering methods (75% threshold) and SRDA models, with Complete Canberra Measure computed on their whole list sets.

In order to get a more refined evaluation of dissimilarity, we also compute the Core Canberra Measures between all Borda optimal lists and between all 75%-threshold partial lists for filtering methods, together with the corresponding partial and Borda lists for the SRDA models: all results are reported in Table 12. By using the Core measures, we draw two levelplots (for both distances on Borda lists and on the whole partial lists sets), computing also a hierarchical cluster with average linkage and representing also the corresponding dendrograms in Fig. 12 and Fig. 13.

**Table 12:** Core Canberra Dissimilarity Measure between Borda optimal lists (upper triangular matrix) and between all partial lists (lower triangular matrix, 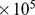) for filtering methods (75% threshold) and SRDA models.

	*F*	*FC*	*modF*	*modt*	*t*	*B*	*SAM*	*SRDA*	F	FC	modF	modt	t	B	SAM	SRDA
*F*	▪	0.007	0.011	0.230	0.115	0.012	0.127	0.010	0.000	0.001	0.003	0.077	0.127	0.003	0.057	0.004
*FC*	**122**	▪	0.016	0.231	0.116	0.018	0.128	0.017	0.007	0.008	0.009	0.084	0.134	0.009	0.064	0.010
*modF*	**69**	**129**	▪	0.228	0.114	0.002	0.126	0.021	0.011	0.012	0.013	0.087	0.136	0.013	0.067	0.014
*modt*	**7324**	**7337**	**7307**	▪	0.165	0.228	0.163	0.239	0.230	0.231	0.232	0.303	0.352	0.232	0.283	0.234
*t*	**2418**	**2441**	**2401**	**7379**	▪	0.115	0.108	0.125	0.115	0.116	0.117	0.192	0.244	0.118	0.173	0.119
*B*	**73**	**132**	**75**	**7308**	**2402**	▪	0.127	0.022	0.012	0.013	0.014	0.088	0.138	0.014	0.068	0.016
*SAM*	**3925**	**3924**	**3912**	**7287**	**4084**	**3914**	▪	0.136	0.127	0.128	0.129	0.201	0.250	0.130	0.181	0.131
*SRDA*	**998**	**1116**	**1067**	**8326**	**3423**	**1071**	**4916**	▪	0.010	0.009	0.012	0.084	0.133	0.012	0.062	0.011
F	**19**	**115**	**63**	**7317**	**2412**	**66**	**3919**	**1004**	▪	0.001	0.003	0.077	0.127	0.003	0.057	0.004
FC	**51**	**159**	**106**	**7360**	**2455**	**110**	**3962**	**976**	**55**	▪	0.004	0.077	0.127	0.004	0.057	0.003
modF	**52**	**111**	**59**	**7313**	**2408**	**63**	**3915**	**1049**	**45**	**88**	▪	0.077	0.127	0.001	0.057	0.005
modt	**1124**	**1216**	**1162**	**8393**	**3478**	**1165**	**4990**	**2032**	**1124**	**1123**	**1126**	▪	0.066	0.078	0.052	0.078
t	**2194**	**2284**	**2229**	**9449**	**4535**	**2233**	**6048**	**3070**	**2194**	**2195**	**2195**	**2081**	▪	0.128	0.094	0.128
B	**60**	**120**	**67**	**7321**	**2416**	**71**	**3923**	**1057**	**53**	**97**	**29**	**1126**	**2196**	▪	0.058	0.006
SAM	**1002**	**1095**	**1041**	**8283**	**3371**	**1045**	**4879**	**1843**	**1003**	**997**	**1004**	**1188**	**2190**	**1004**	▪	0.057
SRDA	**385**	**504**	**455**	**7711**	**2806**	**459**	**4311**	**1015**	**392**	**370**	**436**	**1406**	**2470**	**445**	**1241**	▪

Rows and columns 1–8 (*Italic*): Sweden cohort; rows and columns 9–16: US cohort.

**Figure 14:**
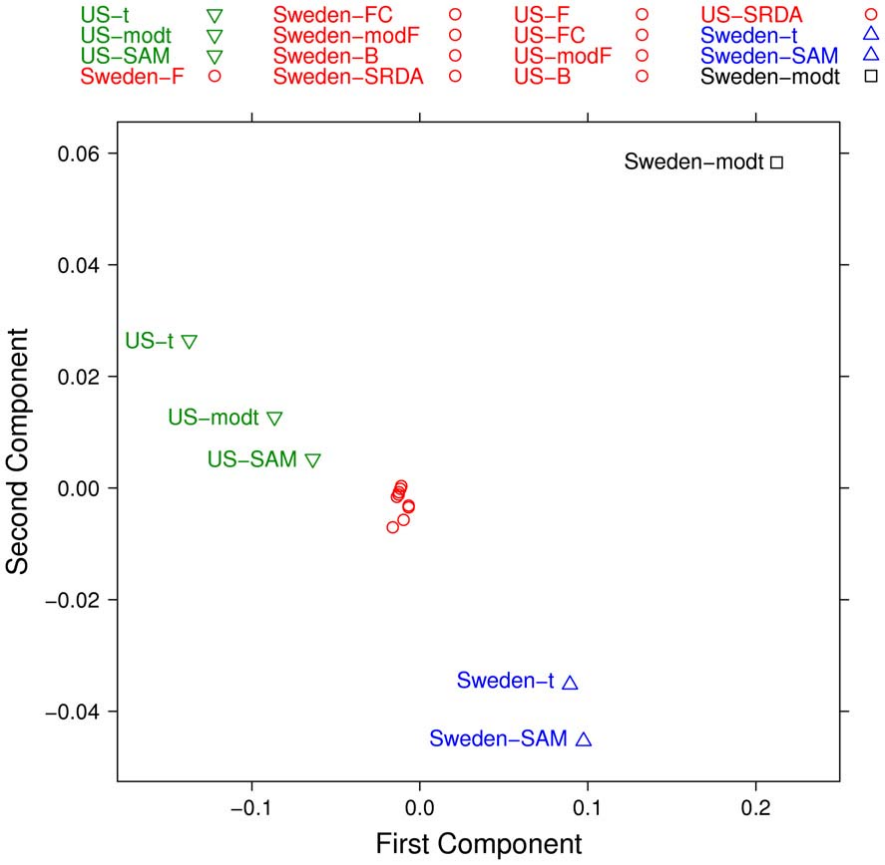
Multidimensional Scaling (MDS) on two components computed on the lists produced by filtering methods (75% threshold) and SRDA models, with Complete Canberra Measure computed on their Borda lists.

**Figure 15:**
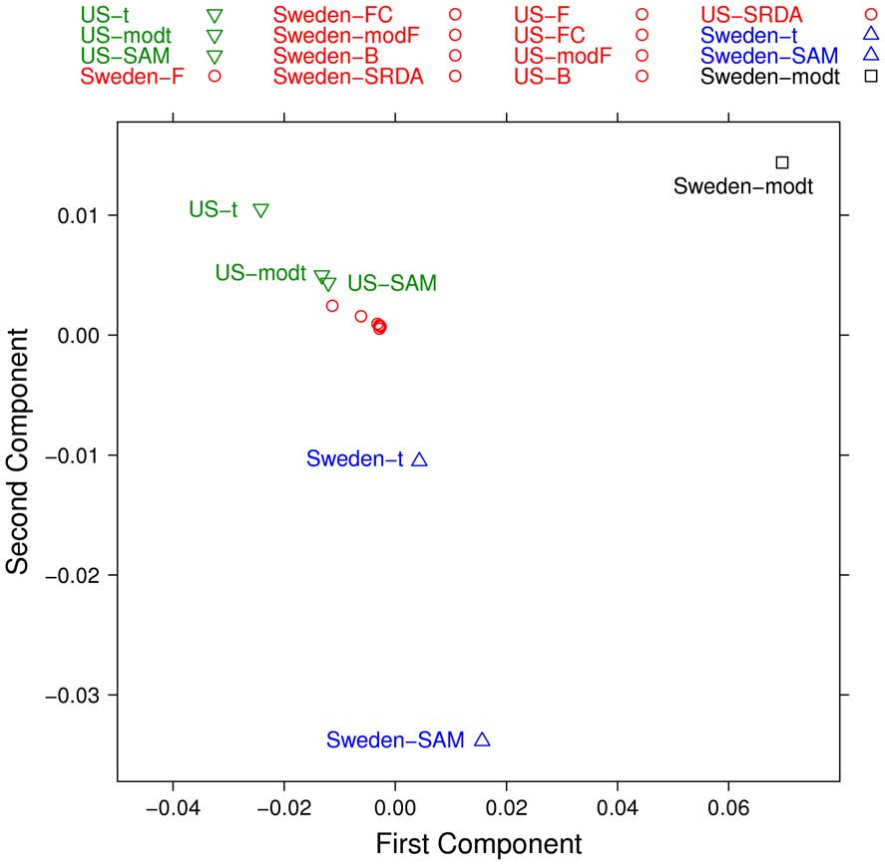
Multidimensional Scaling (MDS) on two components computed on the lists produced by filtering methods (75% threshold) and SRDA models, with Complete Canberra Measure computed on their whole lists.

A structure emerging from the partial list dissimilarity measures has been highlighted by using a Multidimensional Scaling (MDS) on two components, as shown in Fig. 14 and Fig. 15. A few facts emerge: in both cohorts, the results on the Borda lists and on the whole sets of lists are similar, indicating that the Borda method is a good way to incorporate information into a single list. This result confirms the grouping detected by machine learning in the previous subsection. The differences between lists in the two cohorts are quite large, while the lists coming from the profiling experiments are not deeply different from those emerging by the filtering methods.

## Discussion

The research community in bioinformatics requires solutions that accommodate the problem of reproducibility as more and more complex high-throughput technologies are developed. Large scale projects such as the FDA’s MAQC-II analyzed the impact of different sources of variability on the identification of predictive biomarkers [5]. This paper has introduced a partial list analysis procedure that quantitatively assesses the level of stability of a set of ranked lists of features with different lengths. We have shown how to use the Canberra distance in a microarray data analysis study, with application both to multivariate machine learning methods as well as to standard univariate statistical filters. We argue that this is a case of quite large applicability, in which the new method can help select models that have both fair predictivity and stability of the resulting list of biomarkers. Indeed, MAQC-II found an association between predictive performance of classifiers on unseen validation data sets and stability of gene lists produced by very different methods [5].

For bioinformatics, the Canberra distance on partial lists can have a large variety of applications, whenever it is important to manage information from ranked lists in practical cases [1]–[4]. The range of possible applications is clearly wider. At least two additional applications are worth mentioning: first, the approach can be used in the analysis of lists produced by gene list enrichment, as shown in [8] in the complete list case. Second, the most interesting aspect is its extension to more complex data structures, *i.e.*, molecular networks.

As a final consideration, we note that the stability indicator may be used for theoretical research towards a stability theory for feature selection. For classifiers, sound approaches have been developed based on leave-one-out stability [85], [86]. Similarly, our list comparison method could be adopted to build quantitative indicators that can be combined with existing approaches [87]–[91], in a more general framework for feature selection.
